# Health versus income amid COVID-19: What do people value more?

**DOI:** 10.1371/journal.pone.0267004

**Published:** 2022-05-06

**Authors:** Marco A. Palma, Samir Huseynov, Rodolfo M. Nayga

**Affiliations:** 1 Department of Agricultural Economics, Texas A&M University, College Station, TX, United States of America; 2 Agricultural Economics and Rural Sociology, Auburn University, Auburn, AL, United States of America; Institute for Advanced Sustainability Studies, GERMANY

## Abstract

Public efforts to battle COVID-19 have been portrayed as a trade-off between health and the economy in the U.S. public discourse. We investigate how the U.S. general public prioritizes the *health* and the *income* dimensions amid COVID-19 using an incentivized instrument with real monetary consequences. We also employ between-subject information treatments highlighting negative health and income consequences of the pandemic. Specifically, participants have to divide monetary contributions between two charitable organizations representing either the health or the income dimension. An overwhelming majority of participants supports both dimensions, with higher monetary contributions to the health dimension (56%) compared to income (44%), but the difference is not large. Only a small fraction of respondents contributes exclusively to the health (10%) or income (5%) dimensions. Increasing the salience of negative health outcomes of COVID-19 raises differential token allocations in favor of the health-oriented charity. This finding is important since the course of COVID-19 will be shaped by the policies governments implement and how the general public reacts to these policies.

## 1 Introduction

“**Saving lives and saving the economy are not in conflict right now**.”

*32 Top economic policymakers who served the last three U.S. presidential administrations*

COVID-19 is the greatest pandemic of modern times and the biggest humankind crisis since World War II [1]. After the first case was detected in China, over a third of the world’s population are under social distancing and stay-at-home lockdown measures in an attempt to attenuate the spread of the virus (see https://www.businessinsider.com/countries-on-lockdown-coronavirus-italy-2020-3). Although social isolation measures seem to be an effective instrument for reducing the spread of COVID-19 [2–4], they also cause temporary disruption of non-essential businesses, which results in devastating economic losses [5–7]. This unique environment raises questions about a potential trade-off in public efforts in dealing with the two-dimensional nature of the pandemic. Previous studies suggest that pandemics may induce zero-sum behavior when dealing with their consequences [8]. On the one hand, there is public support needed to combat the shortage of personal protective equipment (PPE) and other medical supplies in many places around the world (i.e., the health dimension). On the other hand, displaced workers without a source of income as a result of the stay-at-home directives need public support to endure the loss of employment or income during the pandemic (i.e., the income dimension). In reality, both dimensions constitute a form of a public good with a symbiotic relationship, which tend to mutually enhance each other. Not raising enough resources for the health dimension increases the vulnerability of medical professionals and the general public that can result in more infections and deaths. Not providing enough resources for displaced workers reduces their ability to comply with the stay-at-home directives, thereby increasing their social exposure and the spread rate of the virus. Therefore, the implied trade-off can also be seen as a two-dimensional public good (see https://economicstrategygroup.org/resource/economic-strategy-group-statement-covid19/). Not surprisingly, many countries including the United States, Japan, Germany and others have implemented massive fiscal stimulus packages to combat COVID-19 in these two dimensions. For instance, the U.S. fiscal stimulus bill encompasses $2 trillion to assist households and businesses affected by the COVID-19 (see https://www.congress.gov/bill/116th-congress/senate-bill/3548/text). In fact, preliminary evidence shows that strict and timely public health measures can lead to a better economic recovery [7, 9–11].

The implied trade-off between the health and income dimensions has caused heated political debates around the world. In the United States, there have been numerous public protests from citizens demanding the easing of lockdown directives and reopening the economy [12]. The tensions are further exacerbated by high profile policy discussions regarding the appropriate timeframe to reopen the economy. Analyses from epidemiologists and economists caution that a premature relaxation of lockdown measures can increase the likelihood of a second wave of the pandemic [4, 5]. Constant discussions in the U.S. public discourse about the “trade-off” between the health and income dimensions of COVID-19 resonate as a heavily socio-political polarizing issue [12]. Partisan views create a political connotation around this subject, which could potentially result in a dilution of public support for both causes. Supporters of each cause are also promoting their agenda by exposing the general public to information supporting the potential consequences of each dimension, which in return increases the polarization of the public discourse. An analysis of Google search trends across the United States between March 19th and April 6th of 2020 highlights this tension. In the indicated time period, internet searches for coronavirus had two top related queries in this particular order: “thank you coronavirus helpers” (which was about acknowledging unprecedented efforts of healthcare workers) and “coronavirus stimulus bill” (which was about Government’s fiscal relief package to alleviate the economic consequences of COVID-19). However, it is not clear whether society at large sees these two dimensions as opposing forces having a zero-sum tradeoff relationship. The underline assumption behind the zero-sum tradeoff is that the Health and Income causes are in contradiction, and support to either of them diminishes the effectiveness of the other cause. The zero-sum-tradeoff attitude also implicitly assumes that efforts to alleviate Health or Income issues compete for the same limited resources. Supporting one cause deprives the “competing” cause of the available public funds. Hence the Health and Income dimensions of the public support are irreconcilable.

It is also possible that some individuals see *health* and *income* public goods in a trade-off while holding different implicit marginal rates of substitution in monetary support to the causes (We are grateful to an anonymous referee for highlighting this important nuance). In this study, we explicitly investigate the latter case with an incentivized survey design. We also shed light on whether the general public regards the alleged contradiction as a “trade-off” by eliciting second-order beliefs (i.e., an individual’s beliefs about others’ attitude). Previous research has shown that second-order beliefs are good proxies showing egocentric biases when individuals project their own thoughts to the self-represented views of the societal majority [13].

It is this tension surrounding the perceived “trade-off” between the health dimension and the economy that provides the motivation for our research question. Unlike most previous disasters, the two-dimensional nature of the COVID-19 pandemic offers a unique framework to investigate the allocation of limited public funds when society faces seemingly competing causes that share the same underlying goal. The main objective of our paper is to investigate this alleged tradeoff between public support in the form of monetary contributions to healthcare supplies versus income needs. More specifically, we are interested in capturing the general public’s views on prioritization of the health dimension and the income dimension inherent during the COVID-19 pandemic under the provision of different information conditions. We investigate our research question with an online experiment using an incentivized public contributions mechanism.

We find that the majority of participants provide contributions to both public goods and the difference between the contribution amounts is not large. Our findings are robust to the inclusion of several control variables representing a wide range of socio-demographic characteristics. The results show that household income and practicing social isolation are positively related to monetary contributions to the Health cause. Political affiliation and views on the federal administration are significant predictors of contributions, but the magnitude of their effects is relatively small.

The results of this paper provide information about the general public views on dealing with the perceived health-income tradeoff during COVID-19. Our findings are important since the course of the COVID-19 pandemic will be shaped by what policies governments implement and by how the general public reacts to these policies. Knowing how the public values the health versus the income dimensions examined in this study will help inform policies. This information can be useful for policy implementation related to the management of future emergency outbreaks of similar nature. Thus, the importance of our results goes beyond the context of COVID-19 and provides new policy perspectives for future crises or emergencies with multiple competing interests.

## 2 The online experiment

We conducted a nationwide incentivized experiment in the United States with 586 participants using Amazon-MTurk. Our sample comprises a broad segment of the U.S. population in terms of race, average age (37 years old), and average household size (2.9) (see S1 Table in S1 Appendix). Participants have a diverse income range with $59,000 median effective household income. In the analysis, we divide the household income by the square root of household size and obtain the effective household income for the importance of this measure for making a society-wide inference [14]. In the survey part of the study, we also asked participants to provide their forecasts for the number of Covid-19 cases in the United States one month after the date of the study (April 6, 2020). According to https://www.worldometers.info/coronavirus/, the United States had 1,263,092 confirmed cases on May 6, 2020. On average, participants predicted 1,170,522 (s.e. = 135,573) cases for the United States, and the forecasted number is not statistically different from the actual number of Covid-19 cases (*p*−*value* = 0.49). Therefore, this finding shows that, overall, participants were very attentive and provided their best responses to our study questions.

Rather than simply asking participants about public support views, we incorporate an incentivized experiment with real monetary allocations in order to obtain more accurate public views when money is on the line. In the online experiment, each participant is asked to completely divide 100 tokens (equivalent to $10) between two charitable causes. This enables us to capture other-regarding preferences without the contamination of self-interest. We decided against allowing participants to keep any funds in order to understand preferences for the allocation of public resources. Allowing participants to keep a part of the funds would be highly correlated with income and whether the participant has been economically affected by Covid-19. It may also confound measures of altruism and warm-glow. One of the charitable causes represents the health dimension (i.e., the Health-Charity). The Health-Charity works to reduce the health consequences of COVID-19 by equipping medical professionals with lifesaving medical resources. The Health-Charity delivers protective masks, exam gloves, and isolation gowns to health-care organizations in areas with confirmed COVID-19 cases. The other charitable cause represents the income dimension (i.e., the Income-Charity). The Income-Charity supports hourly workers who have lost their jobs due to COVID-19 and are not able to work and do not have another source of income (see [15] for the importance of using an incentivized donation to a charitable cause as an instrument for eliciting general public views about controversial issues). The allocation of funds is public, in the sense that participants fully allocate all of the tokens between these two causes using a third party’s money. The experiment was incentivized and participants had a 10% chance that their decision will be realized.

The two charities were selected from two existing GoFundMe campaigns that were presented to participants as GoFundMe campaign A (representing the Health Charity) and GoFundMe campaign B (representing the Income Charity) in order to control for potential past knowledge and reputation effects of the charities. Contributions to the Health Charity go directly to the acquisition and delivery of PPEs for frontline healthcare workers. Contributions to the Income Charity go directly into the pockets of hourly workers (including those who rely on tips) who lost their jobs and source of income due to COVID-19. The contributions to each organization were recorded as anonymous (The certificates of the total amount donations to both charities can be accessed through this link: http://samirhuseynov.com/research/certificates.pdf).

Participants are randomly assigned to one of four between-subject information conditions. We constructed our treatments closely representing the primary narrative and bullet points in the public discourse and mass media to realistically document the state of public opinion on both issues. In the control (*N* = 145) participants are only provided with general information about the COVID-19 outbreak without any reference to health or income issues. A Health treatment (*N* = 145) provides additional information about the pandemic’s devastating effects to public health while an Income treatment (*N* = 150) highlights the rise in unemployment and loss of income related to the coronavirus crisis. Finally, a Combined treatment (*N* = 146) includes information provision of the Health and Income treatments combined (The information given for each treatment is available in the S1 Appendix). Since the combined treatment also contains counter-balancing information regarding income issues, we expect health issues will be more salient in the Health treatment. Overall, the treatment assignments allow us to highlight the importance of health and/or income issues the society faces and, in that context, to make individual token donations salient as important support for eradicating the mentioned problems [16].

## 3 Conceptual framework

We present a simple conceptual framework for our experiment [17]. Assume a state of the world with two public goods *G*_*i*,*k*_, *k* ∈ {*H*, *I*}, where *H* represents Health and *I* Income. Each individual *i* is endowed with *ω* that is completely allocated between *H* and *I* (i.e., *ω*_*i*_ = *g*_*i*,*H*_ + *g*_*i*,*I*_, where *ω*_*i*_ ≥ *g*_*i*,*H*_, *g*_*i*,*I*_ ≥ 0). A simple utility function of social preferences is expressed as *U*_*i*_ = *u*(*g*_*i*,*H*_) + *u*(*g*_*i*,*I*_) + *s*(*G*_−*i*,*H*_) + *s*(*G*_−*i*,*I*_), where *G*_−*i*,*H*_ = ∑_*j*≠*i*_ *g*_*j*,*H*_ and *G*_−*i*,*I*_ = ∑_*j*≠*i*_ *g*_*j*,*I*_. Moreover, *u* and *s* represent the individual utilities derived from own and others’ contributions, respectively.

Private contributions of individual *i* to both public causes depend on the importance of the public good *k* for individual *i* (*θ*_*i*,*k*_, where ∑_*k*_ *θ*_*k*_ = 1), the type of information individual *i* receives (*τ*_*i*_ ∈ (⊤_*H*_, ⊤_*I*_, ⊤_*H*&*I*_, ⊤_*N*_)), the contributions of others (*G*_−*i*,*k*_), and other socio-demographic characteristics (*χ*_*i*_) (Λ_*i*,*k*_ = {*θ*_*i*,*k*_, *τ*_*i*_, *G*_−*i*,*H*_, *G*_−*i*,*I*_, *χ*_*i*_}). ⊤_*H*_, ⊤_*I*_, ⊤_*H*&*I*_ and ⊤_*N*_ are mutually exclusive binary variables equal to 1 if *i* is exposed to information favoring the public good *H*, the public good *I*, both public goods, or neither of them, respectively. Then, the utility function can be reformulated as *U*_*i*_ = *u*(*g*_*i*,*H*_(Λ_*i*,*H*_)) + *u*(*g*_*i*,*I*_(Λ_*i*,*I*_)) + *s*(*G*_−*i*,*H*_) + *s*(*G*_−*i*,*I*_).

Assume that the own contribution function $g_{i,k}\left(\theta_{i,k},\Lambda_{i,-\theta_{i,k}}\right)$ is a non-negative and increasing function of *θ* with the following properties: $g_{i,k}\left(0,\Lambda_{i,-\theta_{i,k}}\right)=0$ and $g_{i,k}\left(1,\Lambda_{i,-\theta_{i,k}}\right)=\omega_{i}$. Fixing all other factors (${\bar{\Lambda }}_{i,-\theta_{i,k}}$), we can elicit *θ*_*i*,*H*_ and *θ*_*i*,*I*_ with observed individual contributions $g_{i,k}\left(\theta_{i,k},{\bar{\Lambda }}_{i,-\theta_{i,k}}\right)$ to *H* and *I*. If individual *i* exclusively supports the public cause *H* (i.e., *θ*_*i*,*H*_ = 1; *θ*_*i*,*I*_ = 0), then she will contribute the entire endowment to *H* (*g*_*i*,*H*_(*θ*_*i*,*H*_, ⋅) = *ω*_*i*_ and *g*_*i*,*I*_(*θ*_*i*,*I*_, ⋅) = 0). Conversely, if *i* exclusively supports the public cause *I* (*θ*_*i*,*H*_ = 0; *θ*_*i*,*I*_ = 1), then she will contribute the entire endowment to *I* (*g*_*i*,*H*_(*θ*_*i*,*H*_, ⋅) = 0 and *g*_*i*,*I*_(*θ*_*i*,*I*_, ⋅) = *ω*_*i*_). Higher support for the health cause *H* or the income cause *I* indicates *θ*_*i*,*H*_ > *θ*_*i*,*I*_ or *θ*_*i*,*H*_ < *θ*_*i*,*I*_, respectively. Then, individuals’ contributions will be higher for the cause with the higher support. Finally, having equal support for both causes, *θ*_*i*,*H*_ = *θ*_*i*,*I*_ = 0.5, results in equal contributions, $g_{i,H}\left(\theta_{i,H},\cdot \right)=g_{i,I}\left(\theta_{i,I},\cdot \right)=\frac{1}{2}\omega_{i}$.

If we keep all other factors unchanged (${\bar{\Lambda }}_{i,-\theta_{i,k}}$) via balanced random treatment assignments, and expose individual *i* to information ⊤_*H*_ (⊤_*I*_) favoring public good *H*, then for the same individual contribution amount, the marginal utility of contributing to the public good *H* is greater (less) than the marginal utility of contributing to the public good *I*. When the information provision equally favors both public goods ⊤_*H*&*I*_ (or neutral ⊤_*N*_), then for the same individual contribution amount, the marginal utility of contributing to the public good *H* is equal to the marginal utility of contributing to the public good *I*.

## 4 Main results

**Fig 1:**
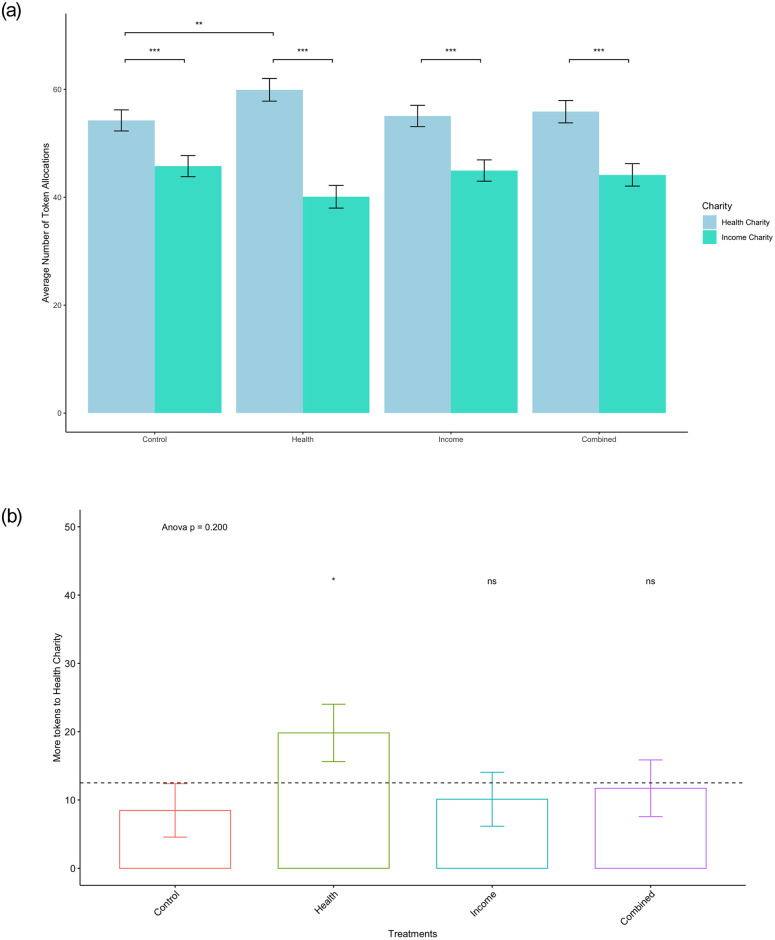
Allocations of tokens between health and income causes. (a) The average number of token allocations for the *health* and *Income* causes. (b) The average number of allocated token differences between the Health and Income causes (positive numbers indicate relatively more allocations to the *Health Charity*). The dashed line indicate the overall sample mean. Standard errors are shown with whiskers. *p < 0.1, **p < 0.05, ***p < 0.01.

Fig 1a shows that the difference in the average number of token allocations between the Health charity and the Income charity across all the experimental conditions is statistically significant (*p*-values < 0.01). On average, participants from all treatments contributed 56 tokens to the health cause and 44 tokens to the income cause. Interestingly, Fig 1b shows that the provision of income information only or the combination of income and health information does not change the health gap contributions relative to the control condition with no information provision. Providing exclusively health information increases the gap from 9 tokens in the Control to 20 tokens in the Health treatment (*p*-value = 0.04). These results suggest that on average, the U.S. public slightly acknowledges the health dimension as the more urgent public problem than the income dimension during this pandemic.

Based on their contribution choices, we define five distinctive types of contributors. Health exclusive and income exclusive contributors use the entire 100 token allocation to support the health and income cause, respectively. These two categories represent the proportion of the sample who exclusively supports one cause. Pro-health and pro-income subjects provide a majority of tokens to their preferred cause. Equal-split participants evenly allocate 50 tokens to each organization.

Shows that Pro-health and Equal-split contributors each constitute around 34% of the sample (68% combined) across all information conditions. Health exclusive and income exclusive contributors are the least observed types across all experimental conditions with an average of 10% and 5%, respectively. Overall there is a very small proportion of participants who reveal a zero-sum trade-off between the two dimensions and contribute exclusively to one of them. We also find that the relative proportion of contributor types only changes in the Health information treatment where there is a larger proportion of Health exclusive types. Notably, providing income information does not change the proportion of income exclusive contributors. All the other contributor types are unresponsive to information exposure treatments. So far, our results suggest that the Health information treatment increases the marginal token allocations by inducing Health exclusive behavior.

Participants were randomly assigned to each information condition and hence our results present a causal effect of information provision on contributions to the health and income causes. However, to further examine the factors that influence contribution allocations, we estimate OLS regressions (see Table 1) with essential socio-demographic indicators. Table 1 presents six models controlling different sets of socio-demographic variables. Table 1 Model 1 tests the impact of our treatment conditions. Recent studies show heterogeneous behavior during the COVID-19 pandemic depending on different demographic factors [18]. Therefore, Model 2 additionally includ es the key demographic variables capturing participants’ age, gender, income, marital status, household characteristics, and the degree of religiosity. Model 3 tests the role of *political affiliation* as the findings of the previous studies document the importance of partisanship in complying with pandemic rules and restrictions [19]. Model 4 and 5 examine the impact of social distancing and isolation measures and individual health conditions on the support of causes, respectively [20]. Table 1 Model 6 combines the previous models to test our treatment effects in the presence of all relevant factors.

**Table 1:** The relationship between differential token allocations to the health charity and individual characteristics.

	*Dependent variable*:					
	Token Allocations to Health Charity—Token Allocations to Income Charity					
	(1)	(2)	(3)	(4)	(5)	(6)
Health Treatment	11.352 **	11.862 **	11.648 **	11.804 **	11.396 **	12.169 **
	(5.763)	(5.798)	(5.736)	(5.766)	(5.790)	(5.843)
Income Treatment	1.638	2.091	1.781	1.619	1.649	2.430
	(5.715)	(5.770)	(5.687)	(5.699)	(5.738)	(5.776)
Combined Treatment	3.243	3.302	2.981	3.373	3.024	3.506
	(5.753)	(5.784)	(5.726)	(5.752)	(5.778)	(5.810)
Gamble choice		-0.483				-0.619
		(1.254)				(1.267)
Female		3.314				2.872
		(4.255)				(4.400)
Age		0.313				0.307
		(0.201)				(0.228)
White		0.234				1.099
		(5.167)				(5.255)
Income		2.098 **				2.027 **
		(1.003)				(1.024)
Income affected moderately		-1.474				-2.768
		(4.506)				(4.569)
Income affected extremely		4.299				3.462
		(6.726)				(6.878)
HH size		0.503				0.515
		(1.164)				(1.237)
Has children		-2.421				-2.665
		(5.709)				(5.885)
Has college degree		0.456				-0.011
		(4.152)				(4.214)
Married		-4.435				-5.516
		(5.162)				(5.306)
Religiosity		-0.904				-0.460
		(0.589)				(0.640)
Political Affiliation			-0.882 ***			-0.698 *
			(0.340)			(0.377)
Practices social distancing				20.119 **		17.351 *
				(9.107)		(9.443)
N. of days stay at home to contain				-0.022		-0.020
				(0.028)		(0.029)
N. of days stay at home for vaccine				0.028		0.014
				(0.029)		(0.030)
Has health insurance					3.593	0.376
					(4.659)	(5.043)
Own Health condition					-3.707	-3.258
					(7.136)	(7.359)
Family health condition					-1.882	-4.137
					(4.583)	(4.757)
Age of oldest adult in HH					0.112	-0.015
					(0.139)	(0.164)
Infected with Covid					-8.506	-1.110
					(13.547)	(14.101)
Knows someone with Covid					8.964	9.327
					(6.407)	(6.468)
Constant	8.469 **	-7.291	6.882 *	-11.334	0.821	-23.255
	(4.075)	(12.241)	(4.101)	(9.604)	(8.231)	(15.460)
Observations	586	586	586	586	586	586
R^2^	0.008	0.033	0.019	0.018	0.014	0.052
Adjusted R^2^	0.003	0.007	0.012	0.008	-0.002	0.009
Residual Std. Error	49.070 (df = 582)	48.960 (df = 570)	48.830 (df = 581)	48.937 (df = 579)	49.179 (df = 576)	48.908 (df = 560)
F Statistic	1.533 (df = 3; 582)	1.282 (df = 15; 570)	2.842** (df = 4; 581)	1.801* (df = 6; 579)	0.890 (df = 9; 576)	1.219 (df = 25; 560)

*p<0.1;

**p<0.05;

***p<0.01

Notes about variables: 1)“Political affiliation” was constructed based on “Approves Rep. Party” and “Approved Dem. Party”—scale [-10,10]. -10—Exclusively supports The Democrat Party and 10—Exclusively supports The Republican Party. 2) “N. of days stay at home to contain” indicates the number of days the participant willing to stay at home to contain the coronavirus. 3)“N. of days stay at home for vaccine” indicates the number of days the participant willing to stay at home to find the vaccine for the coronavirus.

The results of Table 1 confirm Fig 1 and show that, on average, the Health treatment increases the marginal token contributions to the Health cause by around 10 tokens ($1.00). Being more Republican-leaning robustly reduces the size of the gap in token allocations, although the gap is still slightly in favor of the Health cause. Notably, participants who practice social isolation to contain the coronavirus appear to allocate around 17 tokens ($1.50) more to the Health cause compared to the Income cause. For every $10,000 increase in annual effective income the number of tokens allocated to the Health cause increases by around two. The results are robust to different model specifications and to the inclusion of other relevant socio-demographic variables.

The gap in donations favoring the health charity is fairly insensitive to some important socio-demographic covariates such as age, gender, risk preferences (measured through the [21, 22] incentivized lottery choice), religiosity, education, and health status of the respondents or their family. It is notable that the size of the gap is not affected by the participants who experienced a reduction in their disposable income due to COVID-19. Surprisingly, although political views have statistically significant impacts, the magnitude of these effects is not large enough to close the gap in contributions. Practicing social distancing increases the size of the gap in favor of the health dimension. One potential explanation for this result can be that practicing social distancing increases the saliency of the health dimension, hence inducing higher public support to the health cause.

### 4.1 Incentivized beliefs

After making their contributions, participants provide their beliefs about the median contributions to each cause given by the entire sample. In order to ensure that participants provided their truthful beliefs, this question was incentivized with a monetary reward of $1.00 for each correct answer.

Our objective with this task is to quantify how participants’ beliefs about the aggregate behavior of all respondents (i.e., as a proxy for the behavior of society in general) affects their own contributions. There are two potential channels. Participants may conform and follow what they believe the majority would do or they may diverge to provide stronger support to the cause they believe is in greater need.

**Fig 2:**
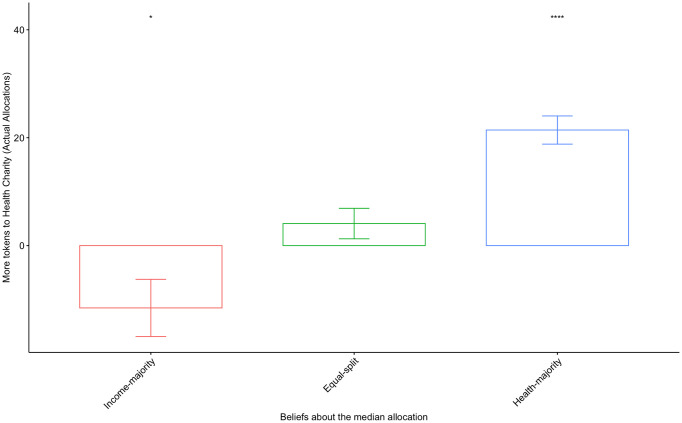
The relationship between beliefs and personal actions. The relationship between the average number of actual allocated token differences between the Health and Income causes and predicted allocations (positive numbers indicate relatively more allocations to the Health cause). The p-value of the ANOVA-test between observed types is less than 0.001. *p < 0.1, **p < 0.05, ***p < 0.01.

**Fig 3:**
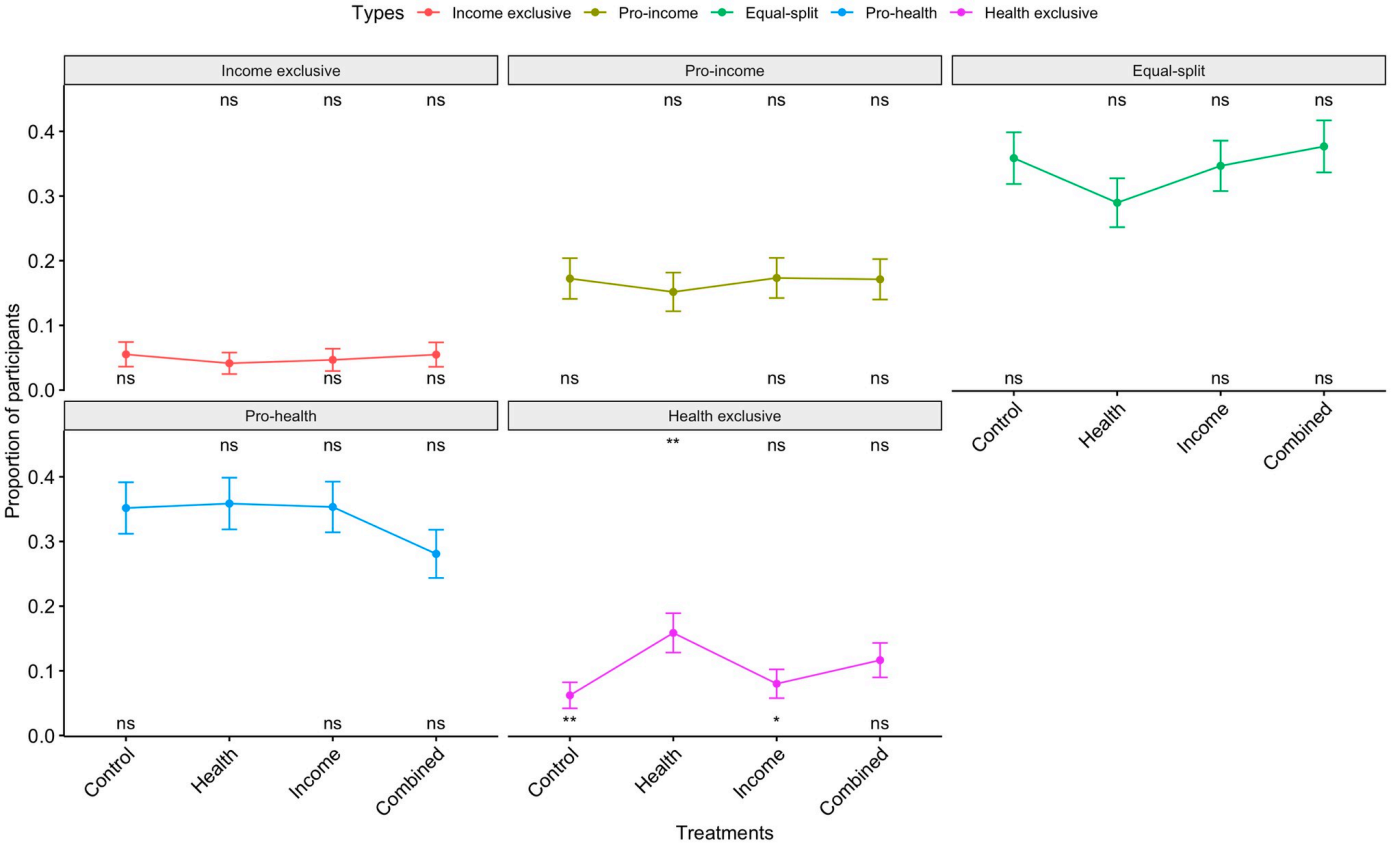
Proportion of participant types across the experimental conditions. The proportion of identified five types across the experimental conditions. *p < 0.1, **p < 0.05, ***p < 0.01.

Fig 2 depicts the results of the incentivized prediction task and its relationship to personal allocations. We identify three belief types based on the prediction of token allocations: 1) Income-majority (16%), for participants who believe that the median allocation will favor the Income cause; 2) Equal-split (20%), for participants who believe that the median allocation will be identical for the Income and Health causes; and 3) Health-majority (64%), for participants who believe that the median allocation will favor the Health cause. Fig 2 shows that there is a strong correlation between beliefs and individual actions. On average, participants allocate their tokens in conformity with their predictions about the contributions of others. S1(a) Fig in S1 Appendix shows that, on average, participants expect higher token allocations to the Health cause relative to the Income cause across all the experimental conditions. So, on average, individual respondents tend to favor the Health cause more than the Income cause and, interestingly, they also believe that their actions are in conformity with societal preferences. S1(b) Fig in S1 Appendix showcases how the relationship shown in Fig 2 changes across experimental conditions. Notably, across all experimental conditions (i.e., Health, Income, and Combined), the information provision treatments induce an increase in the marginal token allocations to the Health cause among participants who also believe that others will allocate more tokens to the Health cause. This result suggests that individuals who believe that public opinion favors health issues during the pandemic become more proactive when treated with conforming or incompatible information. This overlaps with the results shown in Figs 1 and 3, and suggests that Pro-health types are very sensitive to the most salient issue even when the information provided does not necessarily enforce their own priorities.

Overall, our findings do not necessarily imply a causality. It is possible that participants guessed in a way to justify their own allocations. It is also possible that our subjects’ beliefs about others’ actions drove their own token allocations.

## 5 Other important survey results

Our results show that overall, while there is strong support for both health and income dimensions for containing the pandemic, higher support is given to the health dimension than the income dimension. Since the timeframe to resume regular economic activity is at the center of COVID-19 debates, we asked participants how many days they would be willing to remain exercising stay-at-home social isolation measures to control the outbreak. S2(a) Fig in S1 Appendix presents the cumulative distribution of the number of days participants are willing to spend at home exercising social isolation to contain the coronavirus. The median participant is willing to spend around 60 days in social isolation to stop the spread of the virus. S2(b) Fig in S1 Appendix shows that the distribution is sensitive to income level, with lower-income respondents having lower tolerance for social isolation. This result supports the findings presented in Table 1 and shows that the income dimension is more salient to lower-income participants.

Moreover, S2 Fig in S1 Appendix shows that the willingness to remain in social isolation is very sensitive to political affiliation. S2 Fig in S1 Appendix panel (c) shows that there is a significant reduction in the number of days willing to exercise social isolation among pro-republican participants. Overall, our results show that about half of the respondents are willing to practice stay-at-home social isolation for around 30–45 days, and only 25% would be willing to remain in lockdown longer than 100 days.

Social distancing has been considered a key element that can substantially mitigate the impact of COVID-19 [2, 3]. The first wave of COVID-19 has shown that compliance with stay-at-home social isolation requirements is a form of public good [3]. This is especially the case in regions without official enforcement of punishment measures, as is the case in most states in the United States and many other countries. Public outbursts and protests against lockdown directives and business closures are common during the pandemic and based on our survey measures, they are expected to intensify as the number of days in seclusion increases. Thus, our results provide information about the distribution of the support for stay-at-home social isolation directives that can be informative about general public support for this kind of measures.

## 6 Policy implications and conclusion

Public resources to control the spread of COVID-19 are being poured to health and income causes at unprecedented rates. For instance, The United States and Japan have implemented the largest fiscal stimulus packages ever recorded in their countries’ history. In this paper, we study the alleged tradeoff between public support in the form of monetary contributions to healthcare needs versus income support for displaced workers during the COVID-19 pandemic. We investigate this question using an online experiment with real monetary contributions to two charities that focus their fundraising efforts to either the health or income dimension. Our study also introduces an easy-to-apply survey tool that can be used to evaluate future policies as well.

Our results generally imply that the public supports the health dimension slightly more than the income dimension, but that the difference in the level of support is not large. This result is robust to the inclusion of socio-demographic indicators. This finding is likely an acknowledgment of not only the immediate urgency of the health dimension, but also the realization perhaps that the income dimension also has a public health component related to food insecurity, mental health, and stress [23]. Future studies might also use non-online donation field experiments to further investigate public attitude to health and income dimensions of pandemics. Although we cannot disentangle motivations (e.g., pure altruistic, paternalistic, etc.) behind token allocations, our findings provide suggestive evidence on how the public values the health versus the income dimensions provide invaluable policy insights, since pursued government actions and the public reactions to the implemented policies will determine the course of the COVID-19 pandemic. Hence, our main finding reflects the view of the quote at the beginning of this article that “saving lives and saving the economy are not in conflict.”

## Supporting information

S1 Appendix Appendix contains additional supporting information and analyses. (PDF)

S1 Data (CSV)
