# Supplementary material for: Health versus income amid COVID-19: What do people value more?
Source: PLoS One. 2022 May 6;17(5):e0267004. doi: 10.1371/journal.pone.0267004 (PMC9075613; doi:10.1371/journal.pone.0267004)
Supplement: S1 Appendix — (PDF) [file pone.0267004.s001.pdf]

Supplementary Materials

Materials and Methods

The study was approved by Texas A&M University IRB2020-0400M and was pre-registered at AEA RCT registry (https://doi.org/10.1257/rct.5648-1.0).[16] A total of 586 U.S. adults were recruited on Amazon Mechanical Turk platform. Participants were randomly assigned to four experimental conditions: Control (general information about COVID-19 was provided), Health (information about the pandemic's devastating effects to public health was provided), Income (information on the rise in unemployment and loss of income issues related to the coronavirus crisis was provided), and Combined (information from both Health and Income treatments was combined). Each subject received \$5.00 participation fee for participating in the study and was informed that s/he had a chance to earn up to additional bonus \$9.00 in the study depending on her/his decisions or luck. Each participant had 10% to receive the additional payment. After completing the study, each participant was entered into a drawing to be eligible for the additional bonus payment. The computer randomly generated an integer number between 1 and 100. If the random number generated by the computer was between 1 and 10 then the participant received the bonus payment which depended on her/his choices. A total of 57 participants received the bonus payment. No participant was excluded in all four treatments. The socio-demographic characteristics of participants across the treatment were provided in Table S1.

Information treatment conditions.

Control [t0]

Coronavirus COVID-19 The novel coronavirus COVID-19 pandemic is causing devastating effects on the health and wellbeing of people on a global scale. COVID-19 is a disease caused by a contagious new coronavirus. Unlike influenza, there is no known pre-immunity, no vaccine, no specific treatment, and everyone is presumed to be susceptible.

Health [t1]

[16]Before the survey tasks, subjects were shown a consent information form on the screen. Participants were only able to proceed to the experiment after indicating their consent on the screen.

Coronavirus COVID-19 The novel coronavirus COVID-19 pandemic is causing devastating effects on the health and wellbeing of people on a global scale. COVID-19 is a disease caused by a contagious new coronavirus. Unlike influenza, there is no known pre-immunity, no vaccine, no specific treatment, and everyone is presumed to be susceptible.

Scientists estimate that the coronavirus or COVID-19 pathogen could kill between 100,000 and 240,000 Americans. Hospitals across the United States are starting to become overwhelmed by the number of patients seeking treatment due to COVID-19. Medical staff and patient needs are beyond hospital resources and capacities with an unprecedented surge in the number of hospitalized patients.

Doctors and nurses are experiencing shortages in medical equipment supplies. The availability of proper personal protective equipment (PPE) is crucial for the safety and health of medical personnel and the general public. Doctors, nurses, and other health care workers need PPEs to stay healthy to provide treatment to patients with COVID-19 and many other illnesses.

Income [t2]

Coronavirus COVID-19 The novel coronavirus COVID-19 pandemic is causing devastating effects on the health and wellbeing of people on a global scale. COVID-19 is a disease caused by a contagious new coronavirus. Unlike influenza, there is no known pre-immunity, no vaccine, no specific treatment, and everyone is presumed to be susceptible.

The effects of the coronavirus or COVID-19 to the U.S. economy are devastating. Low-income hourly workers and small businesses are expected to feel the largest impact as the global economy will likely go into a recession this year.

Cities across the country are closing down businesses due to COVID-19, causing countless workers to lose their jobs and only source of income. According to the U.S. Bureau of Labor Statistics, only a third of the workforce in the United States is able to work from home. The number of workers' compensation claims has dramatically increased bringing about the potential to increase unemployment rates to even higher levels than the 2008 Great Recession.

Health and Income Combined [t3]

We combined the information presented in the Health and Income treatments and randomized the presentation order.

After reading the provided information in each experimental condition, participants allocated available 100 tokens among the Health and Income charities and also provided their beliefs on the median amount of tokens allocated to each charity after the study (Task 1). The presentation order of the charities was randomized across participants. Participants selected their preferred gambles in Task 2 (See supplementary materials for details). Then participant completed a survey.

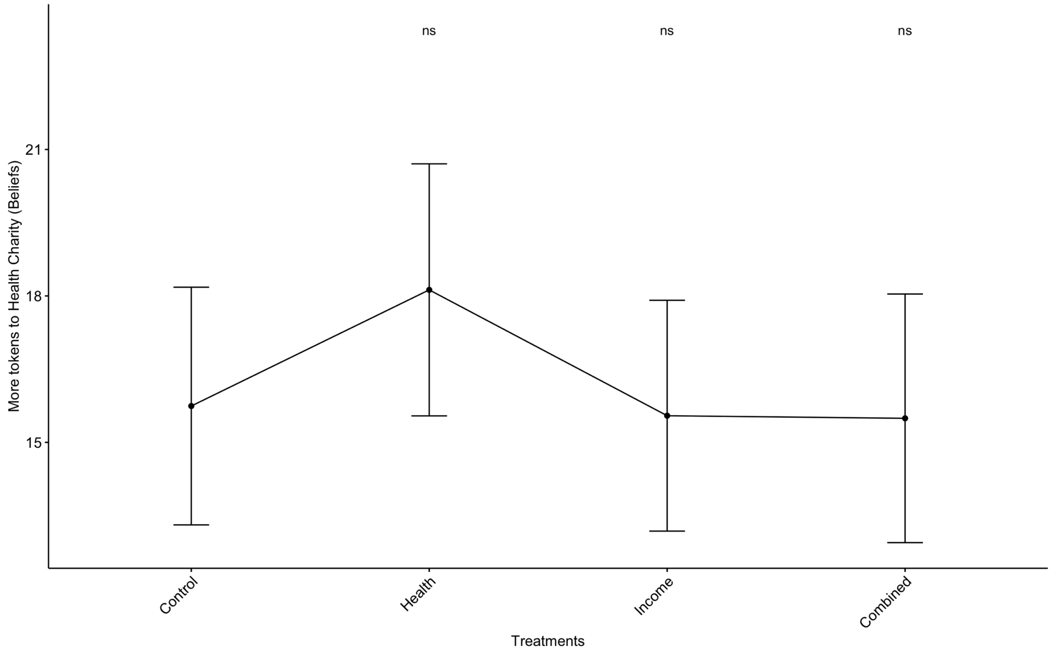

(a)

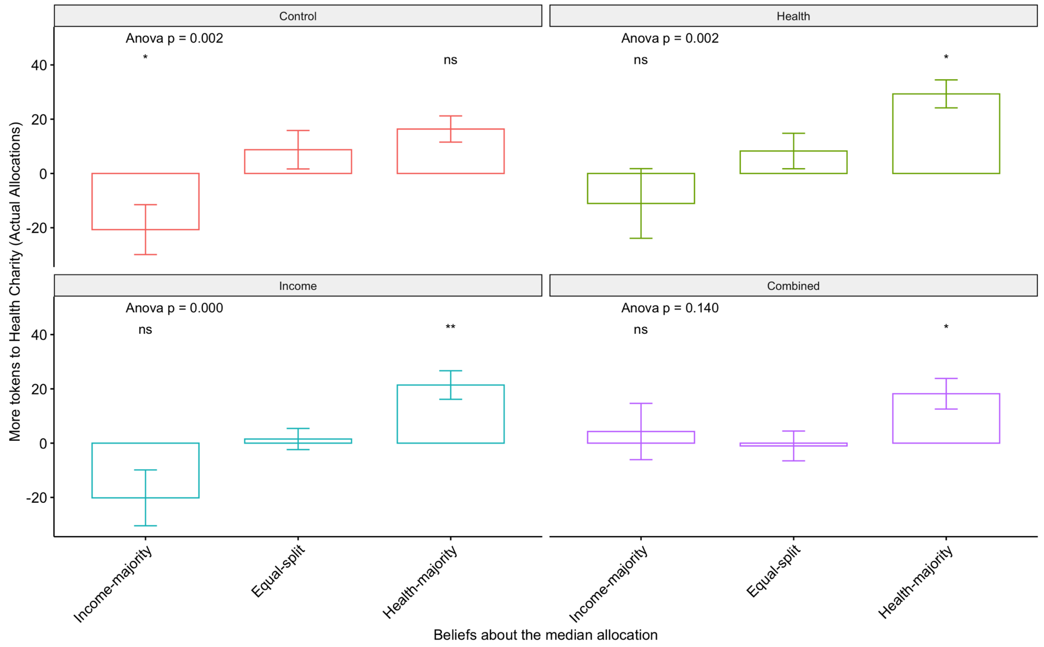

(b)

Figure S1: The relationship between beliefs and personal actions. (a) The average number of predicted token difference (beliefs) between the Health and Income charities across experimental conditions (positive numbers indicate relatively more allocations to the Health Charity). (b) The relationship between the average number of actual allocated token differences between the Health and Income charities and predicted allocations across experimental conditions (positive numbers indicate relatively more allocations to the Health Charity). $*p < 0.1$, $**p < 0.05$, $***p < 0.01$.

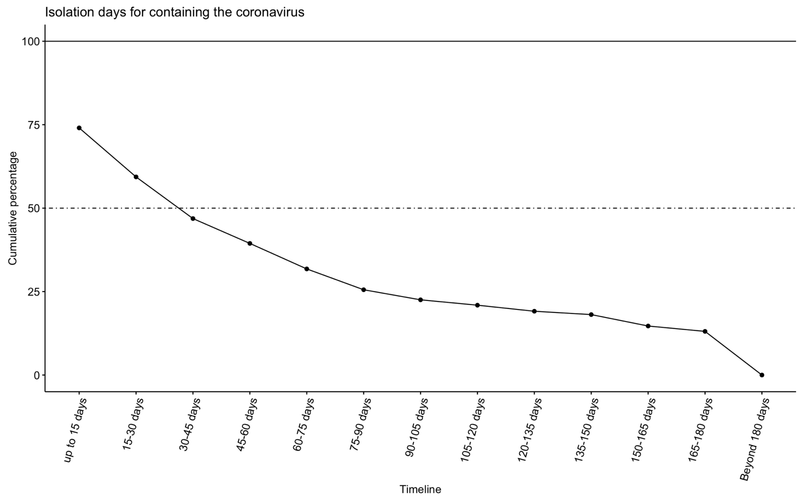

(a)

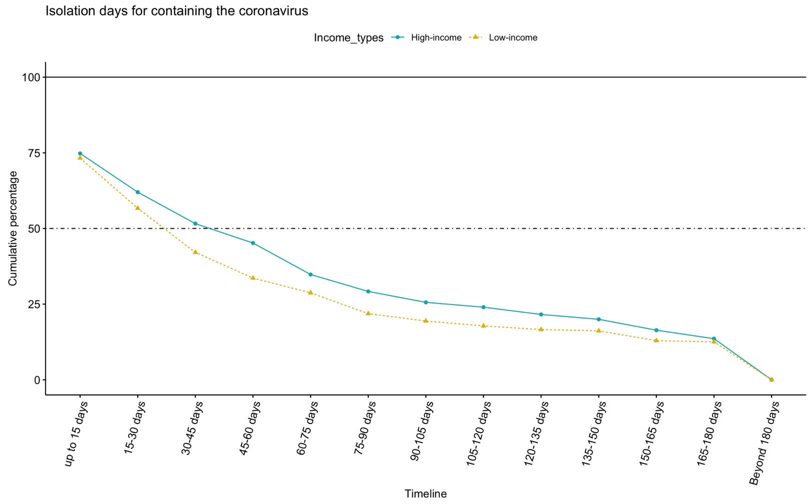

(b)

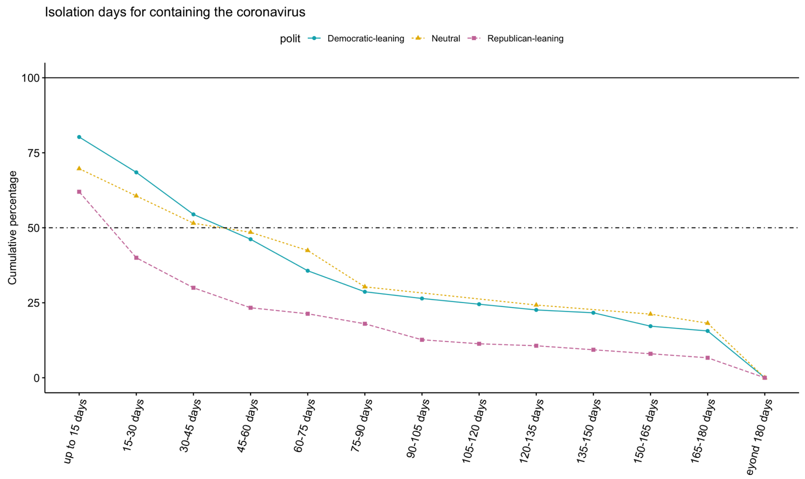

(c)

Figure S2: The relationship between beliefs and personal actions. (a) The cumulative distribution plot of the entire sample. (b) The cumulative distribution plot by income levels. (c) The cumulative distribution plot by political affiliations. $*p < 0.1$, $**p < 0.05$, $***p < 0.01$.

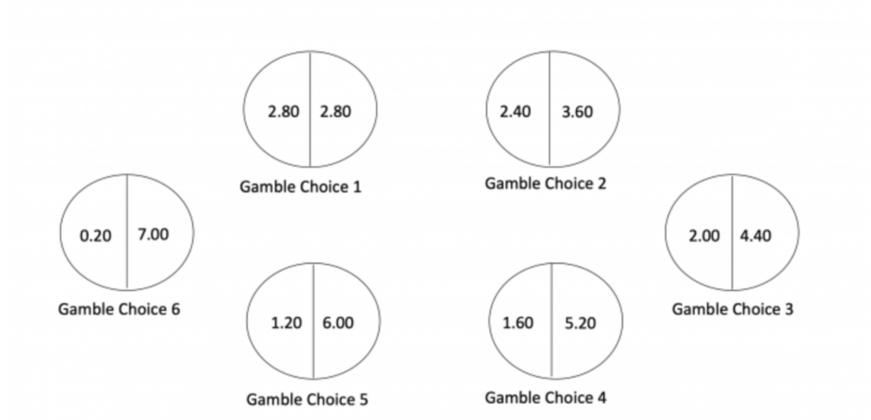

Figure S3: Gamble Choice task

Table S1: The Balance Table of Main Socio-Demographic Variables Across Treatment Conditions

	(1) Control	(2) Health	(3) Income	(4) Combined	(5) (1) vs. (2), p-value	(6) (1) vs. (3), p-value	(7) (1) vs. (4), p-value	(8) (2) vs. (3), p-value	(9) (2) vs. (4), p-value	(10) (3) vs. (4), p-value	(11) p-value from joint orthogonality test
Female	0.35	0.43	0.46	0.38	0.19	0.06	0.66	0.58	0.38	0.15	0.22
	(0.04)	(0.04)	(0.04)	(0.04)							
Age	35.94	36.72	37.43	36.59	0.54	0.26	0.61	0.57	0.91	0.50	0.73
	(0.96)	(0.84)	(0.92)	(0.83)							
White	0.84	0.79	0.79	0.77	0.23	0.29	0.15	0.88	0.80	0.69	0.46
	(0.03)	(0.03)	(0.03)	(0.03)							
Income	37818.53	37874.45	36382.99	39989.91	0.98	0.57	0.42	0.56	0.43	0.15	0.56
	(1911.73)	(1900.29)	(1663.91)	(1902.94)							
Working Days	18.63	20.12	19.19	20.40	0.12	0.57	0.06	0.32	0.75	0.19	0.21
	(0.72)	(0.62)	(0.69)	(0.61)							
Income extremely affected	0.17	0.11	0.11	0.13	0.17	0.14	0.40	0.92	0.61	0.53	0.46
	(0.03)	(0.03)	(0.03)	(0.03)							
Income moderately affected	0.48	0.48	0.46	0.48	1.00	0.79	0.95	0.79	0.95	0.74	0.99
	(0.04)	(0.04)	(0.04)	(0.04)							
Income has not affected	0.36	0.41	0.43	0.39	0.34	0.19	0.58	0.74	0.69	0.45	0.59
	(0.04)	(0.04)	(0.04)	(0.04)							
Has health Insurance	0.72	0.70	0.75	0.77	0.70	0.66	0.33	0.41	0.17	0.58	0.55
	(0.04)	(0.04)	(0.04)	(0.03))							
Own health condition	0.12	0.10	0.10	0.11	0.71	0.64	0.84	0.92	0.87	0.79	0.97
	(0.03)	(0.03)	(0.02)	(0.03)							
Family health condition	0.38	0.29	0.37	0.32	0.11	0.82	0.31	0.16	0.55	0.42	0.34
	(0.04)	(0.04)	(0.04)	(0.04)							
Religiosity	2.90	3.37	3.24	2.97	0.30	0.44	0.88	0.78	0.37	0.53	0.69
	(0.30)	(0.33)	(0.32)	(0.31)							
HH size	2.85	2.93	3.01	2.99	0.76	0.54	0.57	0.73	0.79	0.90	0.93
	(0.21)	(0.17)	(0.17)	(0.13)							
Has children	0.25	0.30	0.29	0.34	0.29	0.46	0.08	0.75	0.48	0.30	0.36
	(0.04)	(0.04)	(0.04)	(0.04)							
Has college degree	0.49	0.41	0.47	0.49	0.16	0.78	0.95	0.25	0.17	0.82	0.45
	(0.04)	(0.04)	(0.04)	(0.04)							
Married	0.36	0.41	0.43	0.44	0.40	0.23	0.17	0.73	0.59	0.84	0.52
	(0.04)	(0.04)	(0.04)	(0.04)							
Age of oldest adult	45.23	44.09	46.63	45.05	0.51	0.44	0.92	0.14	0.58	0.38	0.53
	(1.27)	(1.16)	(1.27)	(1.29)							
Infected with COVID	0.03	0.03	0.03	0.02	1.00	0.96	0.47	0.96	0.47	0.50	0.84
	(0.02)	(0.02)	(0.01)	(0.01)							
Knows someone with COVID	0.16	0.15	0.12	0.14	0.87	0.34	0.60	0.43	0.72	0.66	0.78
	(0.03)	(0.03)	(0.03)	(0.03)							
Practices social isolation	0.96	0.94	0.96	0.97	0.59	0.95	0.75	0.54	0.39	0.79	0.86
	(0.02)	(0.02)	(0.02)	(0.02)							
Practices social distancing	0.96	0.94	0.96	0.97	0.59	0.95	0.75	0.54	0.39	0.79	0.86
	(0.02)	(0.02)	(0.02)	(0.02)							
Days in social isolation	22.28	23.07	91.44	41.00	0.56	0.32	0.29	0.32	0.31	0.48	0.49
	(0.82)	(1.08)	(69.29)	(17.59)							
Approves Rep. Party	35.71	37.26	35.14	33.42	0.71	0.89	0.58	0.62	0.35	0.68	0.83
	(3.00)	(2.96)	(3.04)	(2.87)							
Approves Dem. Party	53.70	51.89	51.51	54.38	0.65	0.58	0.86	0.92	0.52	0.46	0.86
	(2.82)	(2.77)	(2.74)	(2.77)							
Approves Trump	35.22	32.97	35.31	33.23	0.62	0.99	0.65	0.60	0.95	0.64	0.92
	(3.26)	(3.06)	(3.23)	(2.97)							
Approves Trump Covid	36.59	35.83	35.29	33.10	0.86	0.77	0.40	0.90	0.51	0.60	0.85
	(3.11)	(3.02)	(3.10)	(2.79)							
Approves Trump Economy	38.66	35.60	38.07	38.32	0.46	0.89	0.94	0.55	0.51	0.95	0.87
	(3.02)	(2.86)	(2.99)	(3.01)							
Tradeoff	-13.57	-16.65	-16.12	-17.78	0.37	0.43	0.21	0.87	0.74	0.61	0.63
	(2.32)	(2.48)	(2.23)	(2.39)							
N	145	145	150	146							

Standard errors in parentheses. Notes about variables: 1) Female - binary measure (1-"Yes"), 2) Age - age of participant, 3) White - binary measure (1-"Yes"), 4) Income - Effective Income in USD, 5) Working Days - number of workdays in a month, 6) Income extremely affected - the degree own income affected due to COVID-19, 7) Income moderately affected - the degree own income affected due to COVID-19, 8) Income has not affected - the degree own income affected due to COVID-19, 9) Has health Insurance - binary measure (1-"Yes"), 10) Own health condition - binary measure for having health condition (1-"Yes"), 11) Family health condition - binary measure for having health condition (1-"Yes"), 12) Religiosity - increasing scale [0,10], 13) HH size - size of household, 14) Has children - binary measure (1-"Yes"), 15) Has college degree - binary measure (1-"Yes"), 16) Married - binary measure (1-"Yes"), 17) Age of oldest adult - age of the oldest adult in household, 18) Infected with COVID - binary measure (1-"Yes"), 19) Knows someone with COVID - binary measure (1-"Yes"), 20) Practices social isolation - binary measure (1-"Yes"), 21) Practices social distancing - binary measure (1-"Yes"), 22) Days in social isolation - the number of days spent in social isolation, 23) Approves Rep. Party - scale [0,100], 24) Approves Dem. Party - scale [0,100], 25) Approves Trump - scale [0,100], 26) Approves Trump Covid - approves the way Trump manages the pandemic, scale [0,100], 27) Approves Trump Economy - approves the way Trump manages the economy during the pandemic, scale [0,100], 28) Tradeoff - thinks government should prioritize the health of population (-50)/ the economy (50), scale [-50,50].
